# Targeted retail coupons influence category-level food purchases over 2-years

**DOI:** 10.1186/s12966-018-0744-7

**Published:** 2018-11-15

**Authors:** Xintong Guan, Stephen A. Atlas, Maya Vadiveloo

**Affiliations:** 1 Department of Marketing, University of Rhode Island, 7 Lippitt Road, 216, Kingston, RI 02881 USA; 2 Department of Marketing, University of Rhode Island, 7 Lippitt Road, 204, Kingston, RI 02881 USA; 3 Department of Nutrition and Food Sciences, University of Rhode Island, 41 Lower College Road, 201A, Kingston, RI 02881 USA

**Keywords:** Retail purchase quality, Grocery purchases, Longitudinal, Incentives, Dietary pattern, Intervention, Targeted coupon, Healthful food purchase, Difference-in-difference

## Abstract

**Background:**

Targeted coupons strongly influence purchasing behavior and may represent an innovative approach for improving dietary behaviors.

**Methods:**

The retail analytics firm, Dunnhumby, provided secondary retail data containing grocery transactions, targeted coupon exposures, and coupon use for 2500 households over 2-years. The USDA Quarterly At-Home Food Purchasing Database was used to categorize individual foods into 52 categories and combined into 12 food groups. Mixed effects linear models estimated the difference-in-difference effects of coupon exposure on category-level purchase rate/wk. pre- and post-campaign; models also tested effect modification by food category.

**Results:**

Category-level food purchases significantly increased post-campaign. Mean (SD) food purchases/wk. Among exposed households (17.34 (13.08) units/wk) vs. unexposed households (3.75 (4.59) units/wk) were higher (*p* < 0.001). Difference-in-difference effects of coupon exposure showed a higher increase in purchase rate among exposed vs. unexposed households (5.73 vs. 0.67, *p* < 0.001). Food category significantly modified the association between coupon exposure and coupon campaign. Category-level purchase rate among exposed vs. unexposed households was relatively higher in less healthful (e.g. convenience foods) vs. more healthful categories (e.g. nuts) with a 1.17 unit/wk. increase in convenience foods purchase (*p* < 0.001) vs. a 0.03 unit/wk. increase in nuts (*p* < 0.001). Exploratory analyses suggested that price elasticity of food categories for targeted coupons (1.02–2.81) was higher than previous estimates for untargeted coupons.

**Conclusion:**

Across food categories, coupon exposure increased category-level purchase rate, with a relatively larger effect size for less healthful than more healthful categories. Promising results from this preliminary study suggest that experimental research is warranted to determine whether targeting with the explicit purpose of improving dietary quality can more effectively influence diet, and whether it can do so more cost effectively.

**Electronic supplementary material:**

The online version of this article (10.1186/s12966-018-0744-7) contains supplementary material, which is available to authorized users.

## Background

High quality dietary patterns are important for promoting health and preventing chronic disease, yet most U.S. adults have a dietary pattern poorly aligned with the Dietary Guidelines for Americans. National data suggest that more than 75% percent of US adults consume a diet that is low in fruits, vegetables, and other more healthful food groups while simultaneously exceeding recommendations for saturated fats and added sugar [1]. This pattern of purchasing and subsequent food consumption is a primary risk factor for most leading causes of death and disability in the US [2]. Although nutrition education, nutrition labeling, taxes, and bans have been proposed to improve diet quality [3–6], research generally finds that these approaches are costly and have mixed effectiveness [7–10]. Therefore, innovative strategies to shift people’s food purchasing habits are warranted.

Price incentives are increasingly being proposed as potential interventions to promote healthier dietary patterns, and to date, they have enjoyed moderate success [11, 12]. In a meta-analysis of price elasticity, a 10% price increase was associated with a 2.7% to 8.1% reduction in food purchases, with some foods such as sugar sweetened beverages (SSB) particularly responsive to price change [13]. Similarly, Geliebter et al. [14] found that a 50% discount on fruits and vegetables led to three times more purchases per week, a meaningful change that was sustained 4 weeks after discounts were discontinued. Furthermore, a recent systematic review and meta-analysis found that a smaller 10% subsidy was associated with a 14% increase in fruit and vegetable purchases and a 16% increase in other more healthful foods [15]. Evidence from the Healthy Incentives Pilot study further supported the efficacy of subsidies among 7500 low-income households in Massachusetts, and found that a $0.30 cent incentive for every dollar spent on fruits and vegetables resulted in a 0.24 cup increase in fruit and vegetable intake per day during the 12-month study [16, 17]. Despite the modest improvements in diet quality achieved with existing strategies, opportunities exist to further improve population -level dietary quality with greater consideration for cost effectiveness.

Compared to a standardized, “one size fits all approach,” customization is often effective at influencing consumer purchasing behavior because interventions are optimized at the individual level [18, 19]. Customized incentives, unlike uniform incentives, provide different interventions to customers based on their current purchasing patterns, and constitute a key element of many firms’ strategies to influence consumer behavior [20]. For example, when CVS’s loyalty program began using customer purchase history to promote products that each customer was probabilistically more likely to purchase, CVS’s total sales increased 10% within the first year [21]. Despite evidence that targeting incentives is an effective marketing practice to promote long-term profitability, targeted incentives have not been applied toward the goal of promoting more healthful food purchases. Furthermore, existing untargeted interventions generally subsidize only fruits and vegetables, so it is not clear how robustly targeted incentives will influence food purchasing across different food categories.

The objective of this study is to understand the influence of individually-targeted coupons on consumer purchasing patterns among households unexposed vs. exposed to targeted coupons for less healthful and more healthful dietary purchases. Specifically, this study compares the effect of targeted coupon exposures among products that belong to 12 less healthful and more healthful food groups. Using existing panel data, the present study aims to examine: 1) whether targeted coupon exposures affect category-level food purchase rates, and 2) whether the relationship between coupon exposure and product-category level purchases differs between less healthful and more healthful product categories. This project will provide insight into relationship between targeted coupons and food purchases, help identify whether more healthful foods can be encouraged through targeted coupons, and inform how much to incentivize more healthful food relative to less healthful foods. Such information may provide key insights into a novel strategy to promote people’s food purchases in a more cost-effective and sustainable manner in order to improve population-level dietary quality.

## Methods

### Data

De-identified, household-level publicly-available data from a 2-year longitudinal study were obtained from the retail analytics firm, Dunnhumby [22]. Dunnhumby collected information in 2007 on the product transactions, targeted coupon exposures, coupon usage and demographics of a convenience sample of 2500 households who completed 2.5 million item -level transactions in 5 unique stores belonging to a single chain retailor, which were selected to represent nearly all food purchases. The coupons were only redeemable in the 5 stores. All coupon offers were associated with the customer’s past purchase behaviors. Customers were selected to participate in the study based on their propensity to purchase the specific product, brand, or category. Households had varied purchasing habits, coupon usage histories and backgrounds, including age, marital status, household income, composition, household size, home ownership and number of children. In order to examine the effect of coupon exposure on food purchases, the raw data was restructured as follows. Data were analyzed in 2017.

#### Stage 1: Cleaning of raw databases

15.2% of item level transactions containing no food items were excluded.

#### Stage 2: Aggregating food products in all raw datasets

In order to examine the effect of targeted food coupons across categories, 56,009 individual food items were first categorized into 52 categories delineated in the USDA’S Quarterly Food-at Home Price Database (QFAHPD) [23]. Next, kindred categories were combined into 12 food groups (See Additional file 1: Table S1), which included: (1) fruit, (2) vegetables, (3) SSB, (4) non-SSB (including milk), (5) other added sugars, (6) dairy excluding milk, (7) meat, poultry, fish, and eggs, (8) added fats, (9) whole grains, (10) nuts, (11) convenience foods and (12) refined grains.

##### Exposure variable

An indicator variable was created to denote whether a household received (yes/no) any of the 748 food-related coupons over the 2-year study period.

##### Outcome variable

Purchase rate was computed as the average number of items a household purchased per week (# items/(days in period/7)).

##### Other variables

*Food category:* Food category describes which of the 12 categories each household purchased during 2 years.

*Coupon campaign period:* In order to examine change in purchase rate over time, a binary variable was created (pre-campaign /post-campaign) to indicate whether the purchase occurred prior to receiving a coupon or after receiving a coupon. During the study period, households received 30 campaigns, with an average of 31 unique food coupons per campaign. Coupons were sent to customers as part of the campaign with a specified beginning date (day 224- day 659) and end date (day 264- day 730). Therefore, the start date of each coupon corresponded to the date the campaign was initiated. Using the raw data, the earliest validity date (day 223) and the latest expiration date (day 642) were selected to determine the campaign period (pre-campaign, day< =223; post-campaign, 223< day< 642). Because there were few observations after the last expiration date (days 642–730), this analysis exclusively focused on the pre- and post-campaign periods.

##### Data transformation

All raw data was examined for normality. The distribution of food purchases was not a simple parametric distribution, and contained a high proportion of zero-quantity values with a long right tail, potentially biasing statistical analyses [24, 25]. Therefore, in order to estimate changes in rate due to coupon exposure, zero-quantity purchase data was eliminated after validating that dropping this data to normalize the distribution would not bias the sample in the direction of the hypothesis that targeted coupons increase category-level purchase rate.

Additional file 2: Figure S1 compares how dropping zero-quantity purchases influenced mean purchase rates among the unexposed and exposed group in the pre- and post-campaign periods. Dropping zero transactions attenuated the observed relationship between targeted coupon exposure and purchase rate, indicating that an analysis excluding zero-quantity purchases provides a stricter test of whether targeted coupon exposure increases category-level purchase rate. Similarly, Additional file 3: Table S2 presents zero transaction distributions for each food category. Because there was generally a higher prevalence of zero purchases across food categories (excluding SSB) prior to when coupons could be utilized, dropping zeros suppressed the observed relationship between coupon exposure and purchase rate for all foods and inflates estimates for SSB. Therefore, excluding SSB, the estimated parameters can be considered conservative estimates for the relationship between targeted coupons and category-level purchases.

##### Statistical methods

A difference-in-difference analysis was used to examine whether changes in food purchases in the pre- and post-campaign periods among exposed households were significantly different from the pre- and post- purchasing patterns among unexposed households. This approach allowed for assessing whether the changes in purchasing rate were due to targeted coupon exposures rather than other temporal trends that may have influenced the purchasing patterns in each household [26, 27].

Rate of category-level food purchases per week was modeled as a function of coupon campaign period, coupon exposure, food category and their interactions. The results were analyzed via SAS using a two-way and a three-way mixed ANOVA design with campaign period as the grouping variable and coupon exposure as the within-subjects variable. The effect of targeted coupon exposures on the average category-level food purchase rate, across all food categories and all households was first evaluated. The rate of category-level food purchases per week was empirically modeled as a function of coupon exposure in that category, campaign period, and their interaction. Whether categories differed in how targeted coupons influenced category-level purchases was then considered by modeling category-level purchase rate as a function of coupon exposure, campaign period, food category, and two-way and three-way interactions between each of these factors. The interaction between food category, coupon exposure and campaign period tested whether coupons had different effects among different food categories.

In exploratory analysis, elasticity of coupon redemption was also calculated by dividing percent change in quantity by percent change in price to see how customers responded to price reductions through targeted coupons. First, average weekly purchase quantity across all households at the food category level was calculated. Percent change in quantity was calculated by dividing the product category-level difference-in-difference estimates by the number of units purchased by the exposed households in the pre-campaign period. Including only the exposed households in the denominator is a conservative assumption, since exposed households had higher pre-campaign purchases than the unexposed households. Second, the sticker price of each food purchased and the discount price (in dollars or unit of currency), excluding loyalty discounts (i.e. the discount viewable by the consumer) were calculated. This allowed us to calculate the average percent change in price across all items purchased at the category- level when coupons were applied, including transactions with a coupon and transactions without a coupon. For example, if a person received a $0.50 coupon for one product that cost $1, and applied the coupon to only one of two purchases of that product, the average discount for that product would be $0.25, leading to a 25% discount overall.

## Results

Overall, 2,201,815 food transactions including 56,009 unique foods products occurred over the two-year study period from 2003 to 2005 (average of 0.88 food purchasing trips and 8.24 food products per week for each of the 2500 households). In total, 1,746,594 food coupons (748 unique) were sent out and 77,929 (393 unique) were redeemed by targeted households. Of the 2500 households, 1584 received at least one food coupon and 916 households were consistently not exposed to coupons (See Additional file 4: Table S3). Because demographic data were available for roughly 35% of the sample, it was not included in the resulting analysis.

### Coupon exposure and category-level purchase rate

**Fig. 1:**
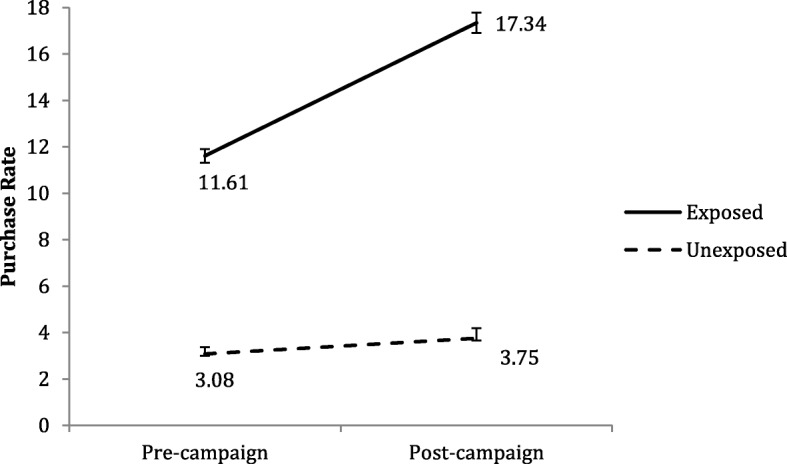
Effect of targeted coupons on purchase rate for all foods. Differences in mean food purchase rate per week pre- and post- a targeted coupon campaign among 2500 households exposed vs. unexposed to a targeted coupon campaign. Exposed households are represented with a solid line and unexposed households are represented with a dashed line. The pre-campaign period describes the period prior to the coupon campaign (day<=223); the post-campaign period describes the period after the coupon campaign began (223 < day< 642)

Table 1 and Fig. 1 show the effect of coupon exposures and campaign period on food purchases among unexposed households and exposed households. The two-way mixed ANOVA yielded significant main effects of coupon exposure and campaign period. In Fig. 1, households exposed to coupon campaigns consistently purchased more food per week than households unexposed to coupon campaigns (*p* < 0.001). Food purchases among unexposed households vs. exposed households also differed significantly in the pre- and post-campaign periods (*p* < 0.001). Mean food purchases per week among unexposed households increased from 3.08 units/week to 3.75 units/week in the post-campaign period. The mean food purchase rate among exposed households was 11.61 units/week in the pre-campaign period and increased to 17.34 units/week in the post-campaign period. The 5.06 units difference-in-difference increase indicated that exposed households purchased 5.06 units more per week than unexposed households in the post-campaign period, relative to each group’s pre-campaign purchase rates (*p* < 0.001).

**Table 1:** Effect of Targeted Coupons on Purchase Rate for All Foods and Each Food Category

Food category^b^	Without coupon exposure		With coupon exposure		Difference In Difference Increase	Significance^a^				
	Pre	Post	Pre	Post		Pre	Post	Unexposed	Exposed	Difference In Difference
	Mean (SD)	Mean (SD)	Mean (SD)	Mean (SD)						
All Foods										
	3.08 (3.63)	3.75 (4.59)	11.61 (9.57)	17.34 (13.08)	5.06	^***^	^***^	^***^	^***^	^***^
Less Healthful foods										
Convenience Foods	0.71 (0.93)	0.86 (1.11)	2.67 (2.50)	3.99 (3.42)	1.17	^***^	^***^	^***^	^***^	^***^
Other added sugar	0.30 (0.46)	0.38 (0.54)	1.12 (1.24)	1.69 (1.64)	0.49	^***^	^***^	^***^	^***^	^***^
SSB	0.36 (0.55)	0.40 (0.59)	1.31 (1.56)	1.81 (2.00)	0.46	^***^	^***^	^***^	^***^	^***^
Refined grains	0.28 (0.52)	0.34 (0.53)	1.01 (1.07)	1.52 (1.38)	0.45	^***^	^***^	^***^	^***^	^***^
Dairy excluding milk	0.22 (0.36)	0.29 (0.51)	0.88 (1.11)	1.37 (1.43)	0.42	^***^	^***^	^***^	^***^	^***^
Added fat	0.04 (0.07)	0.05 (0.08)	0.13 (0.16)	0.22 (0.22)	0.08	^***^	^***^	^***^	^***^	^***^
More Healthful Foods										
Vegetables	0.33 (0.49)	0.42 (0.60)	1.37 (1.42)	2.08 (1.99)	0.62	^***^	^***^	^***^	^***^	^***^
Meat poultry and fish	0.42 (0.56)	0.49 (0.65)	1.53 (1.42)	2.20 (1.82)	0.60	^***^	^***^	^***^	^***^	^***^
Fruit	0.20 (0.31)	0.25 (0.40)	0.82 (0.96)	1.23 (1.35)	0.36	^***^	^***^	^***^	^***^	^***^
Non-SSB	0.17 (0.29)	0.21 (0.42)	0.61 (0.63)	0.95 (0.92)	0.30	^***^	^***^	^***^	^***^	^***^
Whole grains	0.03 (0.02)	0.04 (0.03)	0.10 (0.04)	0.17 (0.01)	0.06	^***^	^***^	^***^	^***^	^***^
Nuts	0.02 (0.06)	0.03 (0.10)	0.08 (0.15)	0.12 (0.22)	0.03	^***^	^***^	^***^	^***^	^***^

Difference in difference analysis. The reference group is households not exposed to coupons. Standard errors were clustered

*SD* Standard Deviation

^*^P < 0.05; ^**^p < 0.01; ^***^p < 0.001

^a^Test of significance between unexposed group v. exposed group in the pre- and post-campaign periods at each food category

^b^ Food categories are grouped from 52 USDA’S Quarterly Food categories including Fresh/frozen fruits; Canned fruits; Fresh/Frozen dark green vegetables; Fruit juice; Canned dark green vegetables; Fresh/Frozen orange vegetables; Canned orange vegetables; Fresh/Frozen starchy vegetables; Canned starchy vegetables; Fresh/Frozen select nutrient vegetables; Canned select nutrients vegetables; Fresh/Frozen other vegetables; Canned other vegetables; Frozen/Dried Legumes; Canned Legumes; Whole grain bread, rolls, rice, pasta, cereal; Whole grain flour and mixes; Whole grain frozen/ready to cook; Other bread, rolls, rice, pasta, cereal, other flour and mixes; Other frozen/ready to cook grains; Low fat milk; Low fat cheese; Low fat yogurt & other dairy; Regular fat milk; Regular fat cheese; Regular fat yogurt & other dairy; Fresh/frozen low fat meat; Fresh/frozen regular fat meat; Canned meat; Fresh/frozen poultry; Canned poultry; Fresh/frozen fish; Canned fish; Raw nuts and seeds; Processed nuts, seeds and nut butters; Eggs, oils, solid fats, raw sugars; Non-alcoholic non-diet carbonated beverages; Non-carbonated caloric beverages; Water; Ice cream and frozen desserts; Baked good mixes; Packaged sweets/baked goods; Bakery items, ready to eat; Frozen entrees and sides; Canned soups, sauces, prepared foods; Packaged snacks; Ready to cook meals and sides; Ready to eat deli items (hot and cold); Non-alcoholic diet carbonated beverages; Unsweetened coffee and tea; Alcohol

### Category differences in coupon exposures and purchase rates

**Fig. 2:**
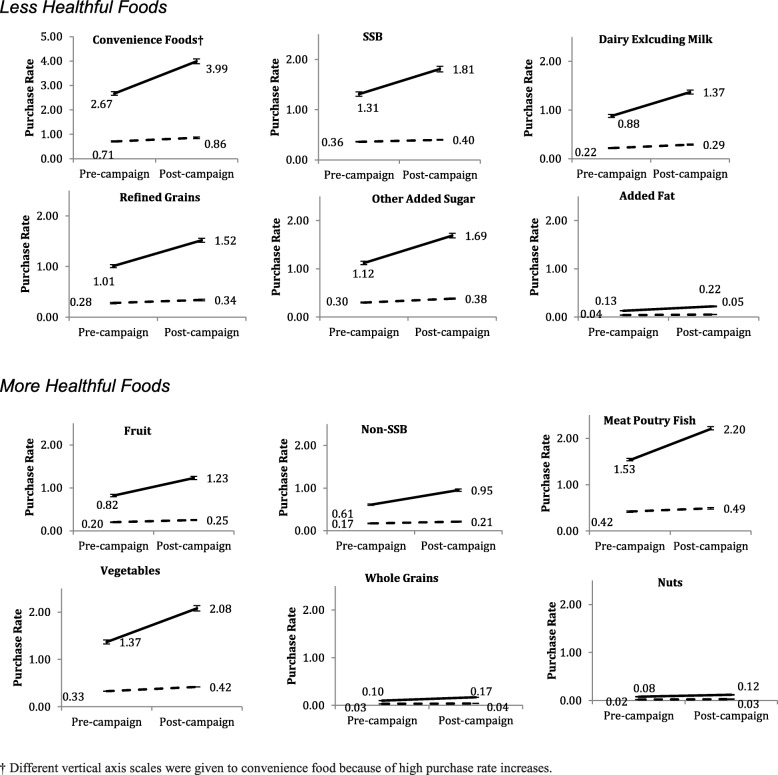
Effect of targeted coupons on purchase rate for each food category. Figures are divided into two groups: less healthful foods and more healthful foods. Mean food purchases per week at food category level among exposed households vs. unexposed households pre- and post-campaign are shown through solid and dash lines. Exposed households are represented with a solid line and unexposed households are represented with a dashed line. The pre-campaign period describes the period prior to the coupon campaign (day<=223); the post-campaign period describes the period after the coupon campaign began (223 < day< 642)

Table 1 and Fig. 2 present differences in purchases rate among all 12 foods categories between unexposed households and exposed households in the pre- and post-campaign periods.

Main effects of coupon exposure and campaign period remained significant in the same direction, with higher food purchases in the post-campaign period (*p* < 0.001) and exposed households consistently purchasing more food per week than unexposed households (*p* < 0.001). Significant three-way interactions among campaign period, coupon exposure and food category were detected, as differences in purchase rate existed among 12 food categories. The greatest increase was among less healthful foods like convenience foods, where the purchase rate among exposed households was 1.17 units greater (*p* < 0.001) in the post-campaign period than the change among unexposed households. The purchase rate of nuts among exposed households was 0.03 units greater (*p* < 0.001) in the post-campaign period than the change among unexposed households, which was lowest among 12 food categories.

In sensitivity analyses, we examined whether coupons encouraged non-purchasing households to start buying a product or whether it only encouraged households who were already buying a product to purchase more. Although our results primarily show that coupons encouraged households who were already purchasing a product to buy more, Additional file 5: Figure S2 shows that the strength of the coupon effect was similar among the smaller proportion of households who were not purchasing a product pre-campaign.

### Elasticity of coupon redemption

Results from the exploratory analysis examining price elasticity are presented in Table 2. Absolute value of coupon redemption elasticity ranged from 1.02 to 2.81, with vegetable purchases least responsive and fruit purchases most responsive to coupons. Households receiving a 10% discount increased vegetable purchases by 10.2% and increased fruit purchases by 28.1%.

**Table 2:** Adjusted coupon redemption elasticity at the food category level

Food Category^b^	Average quantity per unit	%ΔQ	^a^Sticker price per unit	Discount per unit	% ΔP when coupon applied	% purchases using coupon	Adjusted % ΔP	Elasticity
Less Healthful Food								
Added fat	0.22	55%	2.08	−0.78	38%	56%	21%	−2.59
Convenience Foods	3.99	44%	2.24	−0.91	41%	51%	21%	−2.10
Refined Grains	1.69	44%	1.98	−0.94	48%	58%	28%	−1.58
Dairy excluding milk	1.52	45%	1.84	−0.86	47%	62%	29%	−1.54
Other added sugar	1.18	35%	2.34	−1.11	47%	57%	27%	−1.32
SSB	1.37	48%	1.62	−0.85	53%	70%	37%	−1.30
More Healthful Food								
Fruit	1.23	45%	2.12	−0.85	40%	40%	16%	−2.81
Whole grains	0.95	50%	3.08	−1.06	34%	57%	19%	−2.56
Nuts	2.20	44%	2.80	−1.02	36%	62%	22%	−1.97
Non-SSB	2.08	46%	1.65	−0.89	54%	45%	24%	−1.87
Meat poultry and fish	0.12	48%	2.12	−0.93	44%	64%	28%	−1.71
Vegetables	0.17	51%	1.43	−0.99	69%	72%	50%	−1.02

^a^Prices for the food items were discounted from the list price from 3 sources: a loyalty card discount extended to loyalty cardholders, a manufacturer’s coupon paid to the retailer by the manufacturer’s margin, and a retailer match coupon paid out of the retailer’s margin

^b^Food categories are grouped from 52 USDA’S Quarterly Food categories including Fresh/frozen fruits; Canned fruits, Fresh/Frozen dark green vegetables; Fruit juice; Canned dark green vegetables; Fresh/Frozen orange vegetables; Canned orange vegetables; Fresh/Frozen starchy vegetables; Canned starchy vegetables; Fresh/Frozen select nutrient vegetables; Canned select nutrients vegetables; Fresh/Frozen other vegetables; Canned other vegetables; Frozen/Dried Legumes; Canned Legumes; Whole grain bread, rolls, rice, pasta, cereal; Whole grain flour and mixes; Whole grain frozen/ready to cook; Other bread, rolls, rice, pasta, cereal, other flour and mixes; Other frozen/ready to cook grains; Low fat milk; Low fat cheese; Low fat yogurt & other dairy; Regular fat milk; Regular fat cheese; Regular fat yogurt & other dairy; Fresh/frozen low fat meat; Fresh/frozen regular fat meat; Canned meat; Fresh/frozen poultry; Canned poultry; Fresh/frozen fish; Canned fish; Raw nuts and seeds; Processed nuts, seeds and nut butters; Eggs, oils, solid fats, raw sugars; Non-alcoholic non-diet carbonated beverages; Non-carbonated caloric beverages; Water, Ice cream and frozen desserts; Baked good mixes; Packaged sweets/baked goods; Bakery items, ready to eat; Frozen entrees and sides; Canned soups, sauces, prepared foods; Packaged snacks; Ready to cook meals and sides; Ready to eat deli items (hot and cold); Non-alcoholic diet carbonated beverages; Unsweetened coffee and tea

## Discussion

To our knowledge, this is the first longitudinal study using consumer purchasing data to examine whether targeted coupons influence food purchasing patterns and whether differences exist in the strength of this relationship by food category. In this study, households who received targeted coupons significantly increased food purchases, including more healthful foods, more than households who did not receive coupons. Although targeted coupons were not sent with goal of influencing dietary quality, our results revealed that more healthful food purchases, including fruits and vegetables, whole grains, meat fish and poultry, non-SSB, and nuts, were sensitive to targeted coupons. Price elasticities ranged from 1.02 to 2.81, which was notably greater than Andreyeva et al.’s estimates (0.27–0.81), suggesting that people respond more sensitively to targeted coupons than to untargeted coupons.

Nonetheless, it is critical to comprehensively evaluate the feasibility of using targeted coupons to promote the purchase of more healthful foods. To date, most coupons have been applied to unhealthy purchases. In a content analysis of 1056 online store coupons from 6 national grocery chains, researchers noted that snack foods, prepared meals and sodas comprised a large portion of the coupon distribution (41%). In contrast, only 5% coupons were available for more healthful alternatives, such as milk, eggs or yogurt, fresh, frozen or canned fruits and vegetables [28]. However, food shoppers today are becoming more health conscious, and consumers are more interested in dietary improvements that promote health [29]. Thus, companies increasingly need to incentivize more healthful foods to both cater to customers and to have a positive association with their brands- particularly as customers increasingly weigh corporate social responsibility in their purchasing decisions [30]. As consumer demands change, companies will experience pressure to provide monetary incentives for healthful offerings as well in order to increase long-term brand loyalty [29].

Also, targeted coupons may have advantages for increasing dietary quality compared to taxes, bans and uniform incentives. Although more research is needed to understand the effect of targeting coupons to promote more healthful food purchases, and to examine substitution effects, our preliminary results are promising because targeted coupons are theoretically easier to implement, more efficient and sustainable, and more cost-effective. Sugar taxes and bans on less healthful foods are controversial and potentially less effective as they are often perceived as paternalistic and regressive [31]. For example, even though SSB consumption in Berkeley reduced by 10% 1-year following a city-wide soda tax going to effect, sales of SSB in nearby cities rose 7% as people turned to cheaper SSB resources [7]. In school settings, soda bans have similarly had a limited influence on students’ drinking patterns, as students consumed more servings of other soda substitutes such as sports drinks or energy drinks [32]. While less controversial, uniform incentives have generally only experienced modest success and are too costly to implement in the long- term. In the present study, the estimated elasticity for vegetables was 1.02 while fruit was 2.81, suggesting that to achieve same purchasing quantity increase, a lower discount is needed for fruits as fruits are more sensitive to price changes. Such results suggest that targeted coupons are potentially less controversial tools for improving more healthful food purchases in a sustainable and cost-effective manner, warranting further investigation.

Additionally, the potential benefits of targeted coupons are further reflected by their cost-effectiveness when compared against other health promotion programs. A recent systematic review evaluating the cost effectiveness of workplace weight loss programs found that such programs are modestly cost-effective, with a cost ranging from $1.44 to $4.17 per pound of loss in body weight [33]. However, the authors highlighted that a major limitation of existing approaches is that it is not clear whether these interventions reach the highest risk individuals [33], who tend to require more healthcare spending. Based on the higher point elasticities observed in the present study, it is likely that targeting coupons to increase healthful food purchases could be substantially more cost effective by requiring a comparable level of investment while achieving a larger effect size and reaching individuals at the highest risk. Moreover, a recent meta-analysis evaluating the influence of a price decrease on healthful foods estimated that a non-targeted 10% discount on healthful foods would result in a 12% increased consumption of those foods, which would meaningfully influence diet-related morbidity and mortality and associated healthcare costs [15]. In theory, if subsidies on healthful foods are tailored toward the needs of a given individual, the effect size of the intervention and cost savings could improve further, and make it an appealing investment for workplace wellness programs and insurance companies.

Some limitations of the present study must be noted. Demographic data were missing for most households, which limited our understanding of relationships between demographics, coupon exposure and purchase behavior, and may have introduced some selection bias. Additionally, per personal communication with Dunnhumby, most coupons were targeted based on past purchasing behaviors, which suggests that our estimates of the effect of coupon targeting might be confounded with preexisting increases and bias estimates upward; issues pertaining to selection bias should be addressed in future intervention studies. Finally, by dropping observations with zero purchase rates, it is possible to introduce some bias on a category-by-category level. However, sensitivity analyses identified a floor effect and the effect of targeted coupons on food purchases is likely stronger than estimates from the present analysis.

Some strengths of the present analysis are also worth noting. First, this study utilized a unique longitudinal data set to examine the effect of targeted coupons on food purchases across both less healthful and more healthful categories. Companies infrequently release this proprietary information, making it challenging to investigate the effects of targeted marketing in a real-world setting. Additionally, this large sample of 2500 households was monitored over a 2-year period, and generally represented differing income levels, and shopping patterns, which increased the  robustness and generalizability of these findings.

Taken together, additional research examining the effect of targeted coupons is warranted. Future research should explore whether there is additional individual-level variability in responsiveness to coupons across differing household characteristics such as household income, as such information may provide insight about when, how, and how much to use targeted incentives to improve eating patterns among diverse groups- particularly economically disadvantaged households at higher nutritional risk [34]. This may help to develop and refine health-promotion targeting practices by using purchase and survey data to improve individual-level health.

## Conclusion

Public health advocates remain concerned about the high rate of less healthful food purchases due to the association between excess consumption and chronic disease. Existing nutrition interventions are often costly, have mixed effectiveness, or meet consumer resistance, necessitating the adoption of novel strategies to combat less healthful dietary practices. The present study provides promising preliminary evidence that individually- targeted coupons effectively increase category-level food purchases in both less healthful and more healthful categories. The relative cost-effectiveness of this approach warrants further investigation as it may be an efficient and cost-effective lever to improve population-level dietary quality.

## Additional files

Additional file 1: **Table S1.** Food Classification According to USDA’S Quarterly Food Categories. How 52 food categories were grouped into 12 food groups according to Food Classification According to USDA’S Quarterly Food Categories. (DOCX 15 kb)

Additional file 2: **Figure S1.** Comparison on purchase rate between transactions including zero and transactions excluding zero for 2500 households. Differences in mean weekly food purchase rate among exposed households vs. unexposed households with dropping zero transactions vs. without dropping zero transactions pre- and post- a targeted coupon campaign. Transactions excluding zero-quantity purchases are represented with a solid line and transactions including zero-quantity purchases are represented with a dashed line. The pre-campaign period describes the period prior to the coupon campaign (day<=223); the post-campaign period describes the period after the coupon campaign began (223 < day< 642). The supplemental table included in the Additional file 2: Fig. S1 represents ranges of purchase rate between transactions including zero-quantity purchases and transactions excluding zero-quantity purchases. (DOCX 64 kb)

Additional file 3: **Table S2.** Zero Transactions of Each Food Category in pre- and post-campaign periods. Zero transaction distributions for each food category before and during coupon campaign period. (DOCX 16 kb)

Additional file 4: **Table S3.** Descriptive Statistics of Product Information and Coupon Uses. Descriptive statistics of the data including number of households, products, transactions, coupons distribution and coupon redemption. (DOCX 16 kb)

Additional file 5: **Figure S2.** Comparison between households who were already purchasing and households who didn’t purchase before receiving coupons. Additional file 5: Fig. S2 are divided into two parts: Differences in mean food purchase rate per week pre- and post- a targeted coupon campaign among 2500 households who were already purchasing vs. who didn’t purchase before, and differences in mean food purchase per week at food category level among households who were already purchasing vs. who didn’t purchase before. Households who were already purchasing are represented with a solid line and households who didn’t purchase before receiving coupons are represented with a dashed line. The pre-campaign period describes the period prior to the coupon campaign (day<=223); the post-campaign period describes the period after the coupon campaign began (223 < day< 642). (DOCX 534 kb)

## Abbreviations

QFAHPD: USDA’S Quarterly Food-at Home Price Database
SSB: Sugar Sweetened Beverages
