# Supplementary material for: Targeted retail coupons influence category-level food purchases over 2-years
Source: Int J Behav Nutr Phys Act. 2018 Nov 15;15:111. doi: 10.1186/s12966-018-0744-7 (PMC6238299; doi:10.1186/s12966-018-0744-7)
Supplement: Supplementary file 1 — Table S1. Food Classification According to USDA’S Quarterly Food Categories. How 52 food categories were grouped into 12 food groups according to Food Classification According to USDA’S Quarterly Food Categories. (DOCX 15 kb) [file 12966_2018_744_MOESM1_ESM.docx]

**Table S1** Food Classification According to USDA’S Quarterly Food Categories

|  | USDA’S Quarterly Food Categories |
| --- | --- |
| Fruit | Canned fruit; Fresh/frozen fruit; 100% Fruit juice |
| Vegetables | Canned select nutrients vegetables; Fresh/frozen dark green vegetables; Fresh/frozen green vegetables; Fresh/frozen other vegetables; Fresh/frozen select nutrient vegetables; Fresh/frozen starchy vegetables; Frozen/dried legumes; Canned legumes; Canned other vegetables; Canned starchy vegetables |
| Sugar Sweetened Beverages | Non-alcoholic and non-diet carbonated beverages; Non-carbonated caloric beverages |
| Non-Sugar Sweetened Beverages (Including Milk) | Low fat milk; non-alcoholic diet carbonated beverages; Regular fat milk; Non-sweetened coffee or tea; Water |
| Other Added Sugars | Bakery items, ready to eat; Ice cream and frozen desserts; Packaged sweets/baked goods; Baked good mixes; Raw sugars |
| Dairy (excluding milk) | Regular fat cheese; Regular fat yogurt; Other dairy |
| Meat, Poultry, Fish and Eggs | Fresh/frozen low fat meat; Fresh/frozen poultry; Fresh/frozen regular fat meat; Canned fish, Canned meat; Eggs |
| Added Fat | Solid fats; Oil |
| Refined Grain | Other bread, rolls, rice, pasta, cereal; Other flour and mix; Other frozen/ready to cook grains |
| Whole Grain | Whole grain bread, rolls, rice, pasta; Whole grain flours and mixes |
| Nuts | Processed nuts, seeds and nut butter; Raw nuts and seeds |
| Convenience Foods | Canned soups, sauces, prepared foods; Frozen entrees and sides; Packaged snacks; Ready to eat deli items; Ready to cook meals and sides |
