# Supplementary material for: Targeted retail coupons influence category-level food purchases over 2-years
Source: Int J Behav Nutr Phys Act. 2018 Nov 15;15:111. doi: 10.1186/s12966-018-0744-7 (PMC6238299; doi:10.1186/s12966-018-0744-7)
Supplement: Supplementary file 2 — Figure S1. Comparison on purchase rate between transactions including zero and transactions excluding zero for 2500 households. Differences in mean weekly food purchase rate among exposed households vs. unexposed households with dropping zero transactions vs. without dropping zero transactions pre- and post- a targeted coupon campaign. Transactions excluding zero-quantity purchases are represented with a solid line and transactions including zero-quantity purchases are represented with a dashed line. The pre-campaign period describes the period prior to the coupon campaign (day<=223); the post-campaign period describes the period after the coupon campaign began (223 < day< 642). The supplemental table included in the Additional file 2: Fig. S1 represents ranges of purchase rate between transactions including zero-quantity purchases and transactions excluding zero-quantity purchases. (DOCX 64 kb) [file 12966_2018_744_MOESM2_ESM.docx]

**Figure S1** Comparison on purchase rate between transactions including zero and transactions excluding zero

Purchase rate

***Supplemental table** Ranges of Purchase rate between transactions including zero and transactions excluding zero

|  | Excluding zero Transactions | | | | | | Including zero Transactions | | | | | |
| --- | --- | --- | --- | --- | --- | --- | --- | --- | --- | --- | --- | --- |
|  |  | | | | | |  | | | | | |
|  | **Pre-campaign** | | | **Post-campaign** | | | **Pre-campaign** | | | **Post-campaign** | | |
|  |  | | |  | | |  | | |  | | |
|  | Mean | Min | Max | Mean | Min | Max | Mean | Min | Max | Mean | Min | Max |
|  |  |  |  |  |  |  |  |  |  |  |  |  |
| Unexposed | 3.08 | 2.99 | 3.17 | 3.75 | 3.53 | 3.82 | 1.29 | 1.17 | 1.35 | 1.35 | 1.23 | 1.41 |
|  |  |  |  |  |  |  |  |  |  |  |  |  |
| Exposed | 11.61 | 11.31 | 11.91 | 17.34 | 16.90 | 17.78 | 8.50 | 8.17 | 8.77 | 14.56 | 14.09 | 14.97 |
