# Supplementary material for: Targeted retail coupons influence category-level food purchases over 2-years
Source: Int J Behav Nutr Phys Act. 2018 Nov 15;15:111. doi: 10.1186/s12966-018-0744-7 (PMC6238299; doi:10.1186/s12966-018-0744-7)
Supplement: Supplementary file 3 — Table S2. Zero Transactions of Each Food Category in pre- and post-campaign periods. Zero transaction distributions for each food category before and during coupon campaign period. (DOCX 16 kb) [file 12966_2018_744_MOESM3_ESM.docx]

**Table S2** Zero transactions of each food category before and during coupon time

|  | Pre-campaign period | | | | Post-campaign period | | | | |
| --- | --- | --- | --- | --- | --- | --- | --- | --- | --- |
|  | Unexposed | | Exposed | | Unexposed | | | Exposed | |
|  | Zero | Nonzero | Zero | Nonzero | Zero | Nonzero | | Zero | Nonzero |
| Convenience foods | 275 | 662 | 78 | 1485 | 173 | | 764 | 18 | 1545 |
| Dairy excluding milk | 362 | 595 | 126 | 1417 | 239 | | 718 | 35 | 1508 |
| Refine grains | 183 | 743 | 36 | 1538 | 106 | | 820 | 12 | 1562 |
| Other added sugar | 443 | 505 | 264 | 1288 | 300 | | 648 | 72 | 1480 |
| SSB | 272 | 660 | 112 | 1456 | 167 | | 765 | 33 | 1535 |
| Added fat | 805 | 183 | 727 | 785 | 643 | | 345 | 350 | 1162 |
| Fruit | 767 | 206 | 767 | 760 | 616 | | 357 | 355 | 1172 |
| Vegetables | 866 | 103 | 1042 | 489 | 754 | | 215 | 689 | 842 |
| Nuts | 720 | 248 | 647 | 885 | 568 | | 400 | 316 | 1216 |
| Whole grains | 757 | 475 | 377 | 891 | 524 | | 708 | 153 | 1115 |
| Non-SSB | 943 | 2 | 1545 | 10 | 939 | | 6 | 1518 | 37 |
| Meat poultry fish eggs | 745 | 205 | 776 | 774 | 606 | | 344 | 414 | 1136 |
