# Supplementary material for: Targeted retail coupons influence category-level food purchases over 2-years
Source: Int J Behav Nutr Phys Act. 2018 Nov 15;15:111. doi: 10.1186/s12966-018-0744-7 (PMC6238299; doi:10.1186/s12966-018-0744-7)
Supplement: Supplementary file 4 — Table S3. Descriptive Statistics of Product Information and Coupon Uses. Descriptive statistics of the data including number of households, products, transactions, coupons distribution and coupon redemption. (DOCX 16 kb) [file 12966_2018_744_MOESM4_ESM.docx]

**Table S3** Descriptive Statistics of Product Information and Coupon Uses

| Descriptive statistics | Total  n (%) |
| --- | --- |
| Number of households (Households receiving at least one food coupon) | 2,500 (63.4%) |
| Transactions (Transactions containing at least one food item)​ | 2,595,732 (84.8%)​ |
| Products (food) ​ | 92,339 (60.7%)​ |
| Coupon exposures (food coupon exposures) ​ | 1,135 (65.9%)​ |
| Convenience food coupons | 266 (23.4%) |
| SSB coupons | 90 (7.9%) |
| Dairy excluding milk coupons | 76 (6.7%) |
| Refined grain coupons | 105 (9.3%) |
| Other added sugar coupons | 141 (12.4%) |
| Added fat coupons | 35 (3.1%) |
| Fruit coupons | 55 (4.8%) |
| Non-SSB coupons | 60 (5.3%) |
| Meat poultry fish coupons | 137 (12.1%) |
| Vegetable coupons | 113 (10.0%) |
| Whole grain coupons | 9 (0.8%) |
| Nut coupons | 34 (3.0%) |
| Coupon redemptions (food coupon redemptions)​ | 556 (70.7%)​ |
