# Supplementary material for: Targeted retail coupons influence category-level food purchases over 2-years
Source: Int J Behav Nutr Phys Act. 2018 Nov 15;15:111. doi: 10.1186/s12966-018-0744-7 (PMC6238299; doi:10.1186/s12966-018-0744-7)
Supplement: Supplementary file 5 — Figure S2. Comparison between households who were already purchasing and households who didn’t purchase before receiving coupons. Additional file 5: Fig. S2 are divided into two parts: Differences in mean food purchase rate per week pre- and post- a targeted coupon campaign among 2500 households who were already purchasing vs. who didn’t purchase before, and differences in mean food purchase per week at food category level among households who were already purchasing vs. who didn’t purchase before. Households who were already purchasing are represented with a solid line and households who didn’t purchase before receiving coupons are represented with a dashed line. The pre-campaign period describes the period prior to the coupon campaign (day<=223); the post-campaign period describes the period after the coupon campaign began (223 < day< 642). (DOCX 534 kb) [file 12966_2018_744_MOESM5_ESM.docx]

**Figure S2** Comparison between households who were already purchasing and households who didn’t purchase before receiving coupons

***Less healthful foods****:*

***More Healthful Foods:***

† 19 households didn’t purchase any foods before receiving coupons and 2481 households were already purchasing before receiving coupons. 107 households didn’t purchase any convenience foods before receiving coupons and 2393 households were already purchasing before receiving coupons. 789 households didn’t purchase any SSB before receiving coupons and 1711 households were already purchasing before receiving coupons. 213 households didn’t purchase any refined grains before receiving coupons and 2287 households were already purchasing before receiving coupons. 195 households didn’t purchase any added fat food before receiving coupons and 2305 households were already purchasing before receiving coupons. 317 households didn’t purchase any dairy excluding milk before receiving coupons and 2183 households were already purchasing before receiving coupons. 177 households didn’t purchase any other added sugar before receiving coupons and 2323 households were already purchasing before receiving coupons. 305 households didn’t purchase any fruits before receiving coupons and 2195 households were already purchasing before receiving coupons. 162 households didn’t purchase any meat fish and poultry before receiving coupons and 2338 households were already purchasing before receiving coupons. 1254 households didn’t purchase any nuts before receiving coupons and 1246 households were already purchasing before receiving coupons. 232 households didn’t purchase any non-SSB before receiving coupons and 2268 households were already purchasing before receiving coupons. 253 households didn’t purchase any vegetables before receiving coupons and 2247 households were already purchasing before receiving coupons. 1122 households didn’t purchase any whole grains before receiving coupons and 1378 households were already purchasing before receiving coupons.
